# Can homogeneous, lipomatous tumors be primarily resected without biopsy? A retrospective analysis of 240 tumors

**DOI:** 10.1186/s12957-022-02665-4

**Published:** 2022-06-08

**Authors:** Tobias M. Ballhause, Sebastian Weiss, Alonja Reiter, Karl-Heinz Frosch, Andreas M. Luebke, Peter Bannas, Carsten W. Schlickewei, Matthias H. Priemel

**Affiliations:** 1 Department of Trauma and Orthopedic Surgery, University Medical Center Hamburg-Eppendorf, Hamburg, Germany; 2 Department of Trauma Surgery, Orthopedics and Sports Traumatology, BG Hospital Hamburg, Hamburg, Germany; 3 Department of Pathology, University Medical Center Hamburg-Eppendorf, Hamburg, Germany; 4 Department of Diagnostic and Interventional Radiology and Nuclear Medicine, University Medical Center Hamburg-Eppendorf, Hamburg, Germany

**Keywords:** Lipomatous tumors, Soft tissue tumors, Sarcoma, Atypical lipomatous tumor, Liposarcoma

## Abstract

**Background:**

According to guidelines, every soft tissue tumor (STT) larger than 3 cm should be biopsied before definitive resection. Advances in magnetic resonance imaging (MRI) improve the possibility to give a provisional diagnosis of the tumor’s entity. Can lipomas and atypical lipomatous tumors (ALTs) of the extremities therefore be primarily marginally resected based on interpretation of MR images without a previous biopsy?.

**Methods:**

In this retrospective, single-center study, 240 patients with the suspicion of a lipomatous tumor in MRI and surgical treatment in our institution between 2011 and 2020 were included. MR imaging was performed before surgery. All resected specimens underwent histopathological analysis.

**Results:**

The collective comprised 142 tumors that were suspected as lipoma or ALT by the radiologist and underwent primary marginal resection (PMR). One case had myxoid liposarcoma that was underestimated on MRI and needed radical follow-up resection. One-hundred forty-one patients were cured after PMR. Ninety-eight patients were biopsied initially and in 93 cases resected afterwards according to the necessary oncological margins.

**Conclusion:**

In our institution, PMR is performed if a lipoma or ALT is suspected on MR imaging. Our treatment method and the diagnostic algorithm are presented. Primary resection spares patients from one surgical procedure, but a slight risk for underestimation of the tumor remains.

## Background

Lipomatous tumors are the most common among soft tissue tumors (STTs) [1]. They can be found superficially just below the skin or deeper in the soft tissue [2]. In this very heterogeneous group of tumors, the most frequent are lipomas with an incidence of 2100 per 100,000 persons [3]. Lipomas are benign STTs.

Another frequent entity of adipocytic tumors is the atypical lipomatous tumor (ALT). Atypical lipomatous tumor and well-differentiated liposarcoma (WDLPS) are morphologically and genetically identical. However, for neoplasms that arise in the limbs or on the trunk, the term ALT is used, because complete excision is usually curative and achievable. ALT and WDLPS do not have potential for metastases unless they undergo dedifferentiation [4]. When located in deep anatomical regions like the retroperitoneum, spermatic cord, or mediastinum, complete resection can be difficult. The residual tumor potentially relapses and undergoes dedifferentiation, which results in a significantly poorer prognosis [5]. Therefore, in these locations, the tumors are named WDLPS. The treatment method for lipomas and ALTs of the trunk and in the extremities is alike: marginal resection of the tumor with its pseudocapsule [6].

Malignant STTs have an incidence of 4.7 per 100,000 persons [7]. Thus, they only account for 1% of all malignant tumors in adults [8]. At least 70 subtypes of soft tissue sarcomas exist [9]. The three most frequent are undifferentiated pleomorphic sarcoma, liposarcoma (LPS), and leiomyosarcoma [10]. LPS can further be subclassified in well-differentiated liposarcoma (WDLPS), myxoid LPS, pleomorphic LPS, dedifferentiated LPS, and myxoid pleomorphic liposarcoma [11]. Five-year survival of LPS ranges from 50 to 65% in Europe [12]. The wide range is in respect to the various subentities. LPS are treated with wide resection. Their response to chemotherapy is rather poor [13]. However, before resection, an interdisciplinary tumor conference is advised to discuss the individual therapy and a multimodal approach [14, 15].

**Fig. 1:**
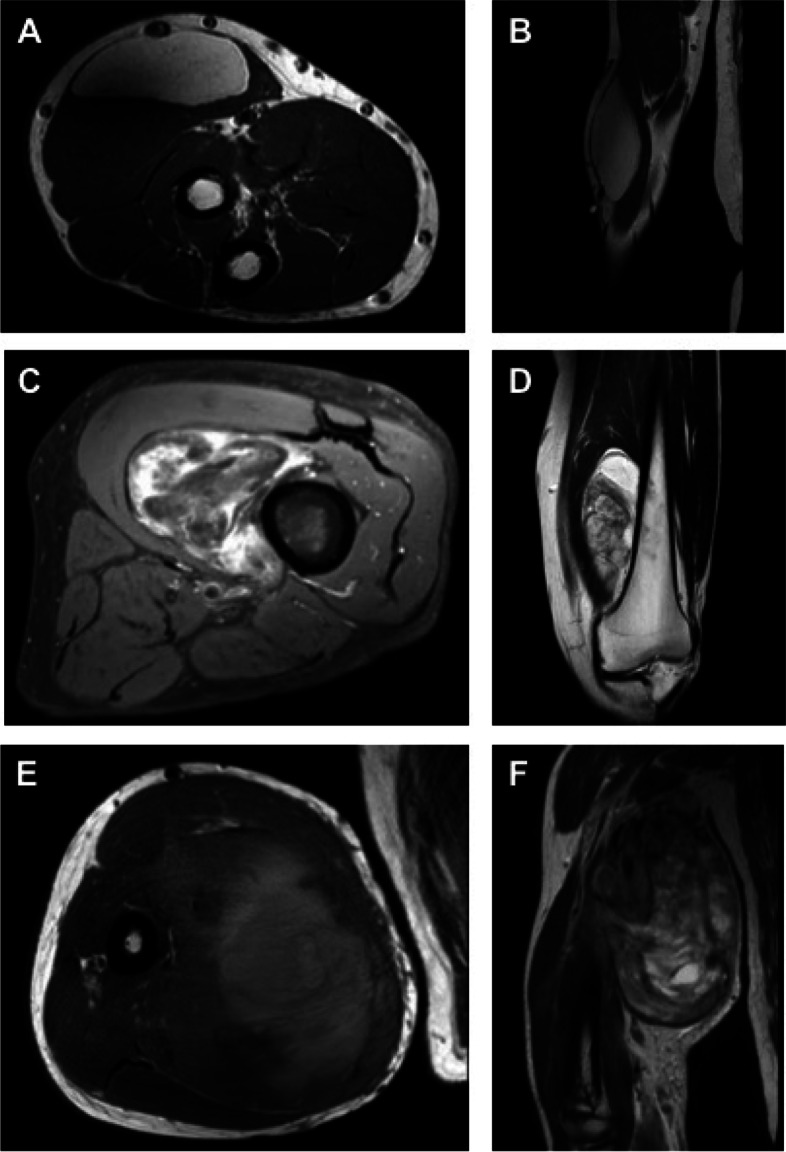
Examples for lipomatous tumors in MRI. **A** Lipoma in a T1-weighted sequence in transversal plane. **B** Corresponding T2-weighted coronal image. **C** Atypical lipomatous tumor (ALT) in a contrast-enhanced fat-saturated T1-weighed sequence in transversal plane demonstrating inhomogeneous contrast enhancement. **D** Corresponding T2-weighted coronal image. **E** Pleomorphic liposarcoma in a T1-weighted-sequence in axial plane. **F** Corresponding T2-weighted coronal image

**Fig. 2:**
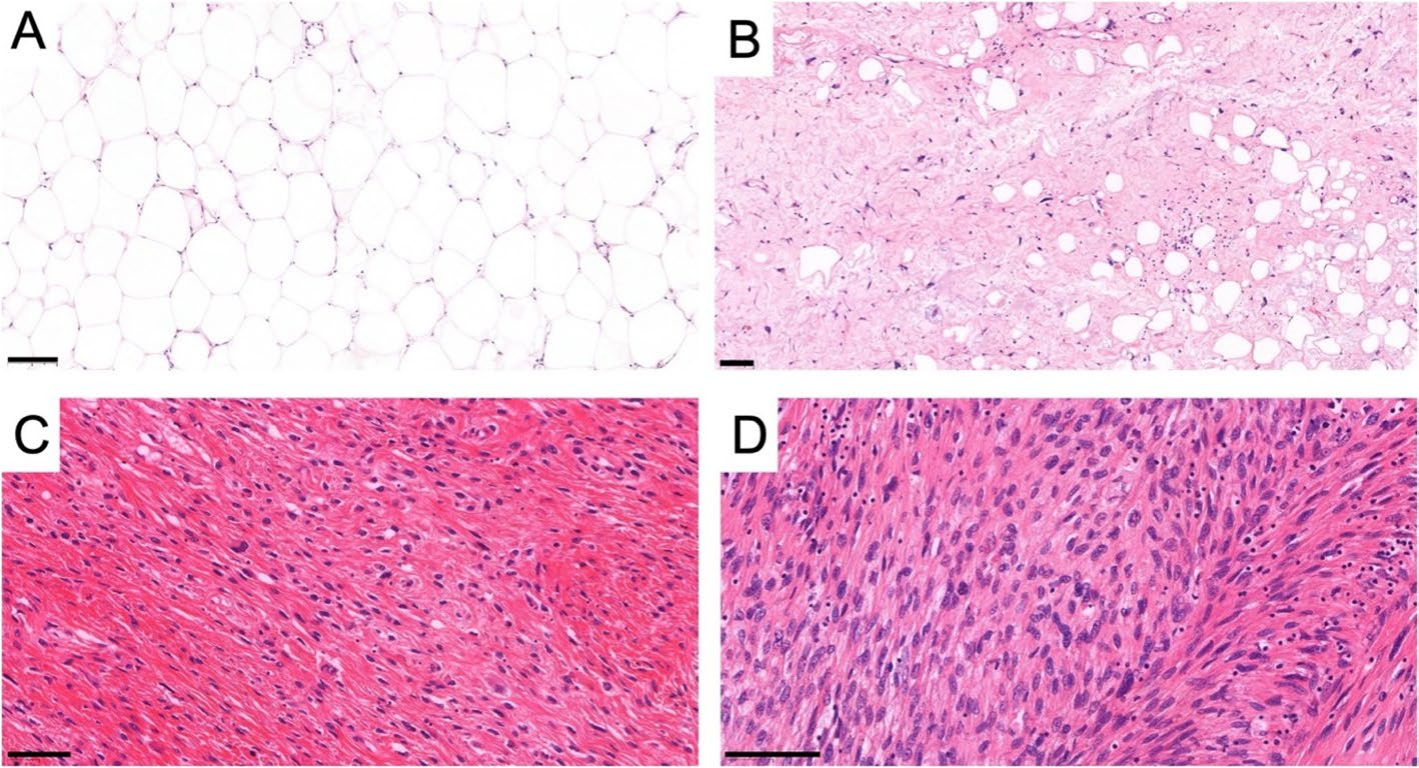
Histologic images of lipomatous tumors. **A** The image shows the characteristic histology of lipoma: a homogeneous proliferation of mature adipocytes without cellular atypia. **B** The adipocytic (lipoma-like) type of atypical lipomatous tumor (ALT) consists of adipocytes with variation in size and shape and a typical component of stromal spindle cells with hyperchromatic atypical nuclei. **C** Liposarcomas with low-grade dedifferentiation are rare but recognized increasingly. The tumor frequently shows dense proliferation of uniform spindle cells with mild nuclear atypia arranged in a fascicular pattern. **D** The dedifferentiated areas of high-grade dedifferentiated liposarcoma usually resemble undifferentiated pleomorphic sarcoma or myxofibrosarcoma. However, any type of high-grade sarcoma can be present. The actual image shows high-grade spindle cell sarcoma with features of fibrosarcoma. All images are in hematoxylin and eosin stain. The black bar indicates 100 μm

Magnetic resonance imaging (MRI) is the most effective diagnostic tool for soft tissue imaging [16]. Figure 1 gives an exemplary overview of the discussed lipomatous tumors on MRI. MRI-based criteria have been developed to assess the tumor’s degree of malignancy [17, 18]. Homogeneity of the MRI signal, especially in T1-weighted sequences with fat suppression, is an indicator for lipomas [19]. The main MRI characteristic that helps to distinguish between lipoma and ALT is the broader and more nodular fibrous septa in the latter [20]. However, neither a clinical nor a radiologic differentiation between lipoma and ALT is safely possible [21]. The state-of-the-art method to distinguish benign lipomas from ALTs is fluorescence in situ hybridization (FISH). In contrast to lipomas, ALTs show mouse double minute 2 (MDM2) amplification [22]. As in all cancerous disease, the final diagnosis is provided by histologic analysis (Fig. 2). Contrast medium (gadolinium) should be applied for every patient who undergoes MRI for soft tissue tumor diagnostics. Malignant lipomatous tumors usually show heterogeneous contrast-medium enhancement [23].

According to guidelines, every tumor should be biopsied before its definitive resection. An exclusion are tumors smaller than 3 to 5 cm in diameter, which can be safely excised [24]. The range in tumor’s diameter is in respect to the various national guideline, which differs to some extent [25]. Yet, these small, unnamed tumors need to be resected *in sano*, to achieve an R0 resection margin. This general recommendation has improved the oncologic outcome of patients and allows to give patients the advantages of targeted, neoadjuvant chemotherapy [26]. Over the course of the past decade, continuous progression of MRI quality and improved prognostication of tumor entity has been made. This raises the question whether tumors with a homogeneous, lipomatous appearance in MRI, in the extremities, can safely be resected without previous biopsy.

In this article, we describe our treatment method for lipomas as well as ALTs and analyze our collective of treated patients. MRI and histological reports were compared to each other. The patients’ charts were searched for the necessity of a follow-up resection after primary marginal resection of a lipomatous tumor.

## Methods

**Fig. 3:**
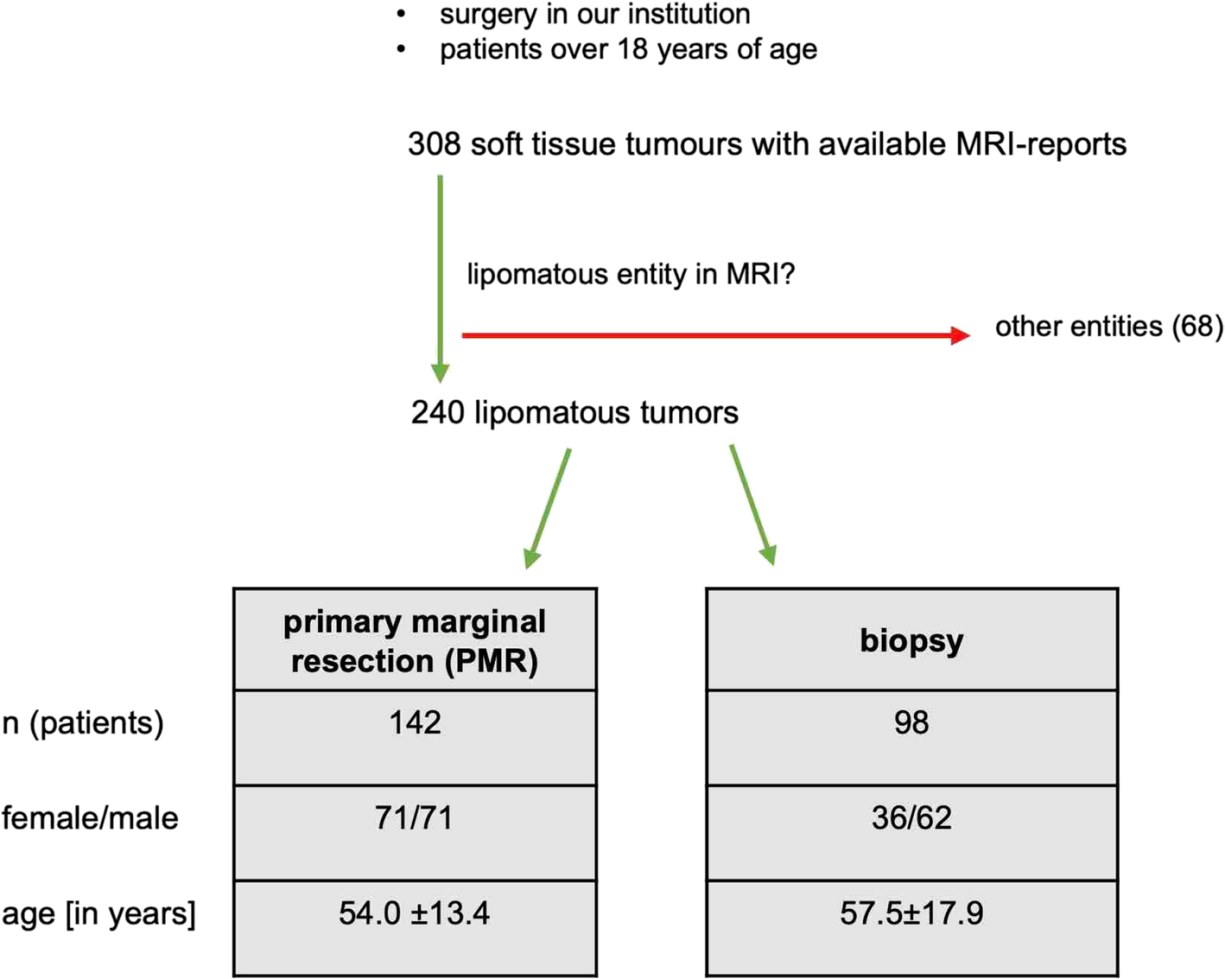
Inclusion criteria. The flow chart depicts the inclusion process of the study and the demographic data of the collectivity

The study had a retrospective, single-center design. Approval was obtained from the institutional review board (WF-071/20). The patients analyzed were surgically treated between 2011 and 2020 at our institution. The inclusion criteria are outlined in a flow chart (Fig. 3). All biopsies were performed as open incisional biopsies. According to the working procedure for STT in our institution, every patient with an STT receives an MRI of the tumorous region, which should be supported by the application of gadolinium [27]. In our patient collective, 93% of the patients received contrast medium. All MRI reports were written by fellowship-trained radiologists. A total of 86% of the MRI scans were not performed in our institution. Consequently, the evaluation of the MRI was done by external radiologists. Their MRI reports were scanned into the patients’ electronic medical charts at our institution. Most patients contacted the sarcoma outpatient clinic of our institution after referral by a resident doctor. Patients were clinically examined, and the MR images were analyzed by two senior surgeons who both have more than 10 years of experience in musculoskeletal tumor surgery. Then, patients received a recommendation for PMR or incisional biopsy of the lipomatous tumors.

All surgical specimen were analyzed by fellowship-trained pathologists in our institution. In cases of doubt, a reference pathology institution was contacted. Lipomas were distinguished from ALTs by FISH for the MDM2 gene [28]. In cases of doubt, tissue samples were sent to an appropriate reference pathology institution. All intrathoracic and intra-abdominal lesions were excluded from the study since different oncology treatment applies to them.

A retrospective comparison of the provisional diagnosis given in the MRI report with the final histopathologic diagnosis was performed. In the case of a biopsy before resection, the histopathologic result from the biopsy was compared to the final analysis of the completely resected tumor.

The study data were statistically analyzed (GraphPad Software 9 Los Angeles, CA, USA). Parametric distribution of the data was tested with the Shapiro-Wilk test. For all data, nonparametric distribution was applied; thus, comparisons were done with Whitney-Mann *U*-test (confidence interval of 95%). A *p*-value less than 0.05 was regarded as significant and less than 0.005 as highly significant.

## Results

Overall, 240 lesions in 240 patients with a suspicion of a lipomatous tumor on MRI were included in the study. One-hundred forty-two (59%) were selected for primary marginal resection. In 98 (41%) of the 240 lesions, an open incisional biopsy was favored instead of primary resection (Fig. 3).

In the cohort of PMR, patients’ average age was 54.0 ± 13.4 years. There were 71 women and 71 men in the cohort of PMR. After resection, histopathologic analysis of the specimens revealed 116 (81.7%) lipomas, 25 (17.6%) ALTs, and one myxoid LPS (0.7%) (Table 1). For these 142 tumors, the concordance of the provisional diagnosis in the radiologists’ MRI report with the final histopathologic diagnosis was 74%. In 25%, the radiologist’s provisional diagnosis overestimated the tumor’s degree of malignancy. Only in one case a tumor was falsely diagnosed as ALT in the MRI report, but histology revealed a myxoid LPS. MRI was performed with use of contrast medium. This malignant tumor needed a radical follow-up resection. The affected patient was a 65-year-old male. The myxoid liposarcoma measured 200.7 cm^3^, which is smaller than the average LPS in the study (706.6 cm^3^). It was located in close relation to the musculus latissimus dorsi. Two weeks after the first surgery, a radical oncological follow-up resection was performed.

**Table 1:** Histologic diagnoses of the primarily resected lesions. In total, 142 tumors were treated with primarily marginal resection. The table depicts the final histopathologic diagnosis of all resected specimens

Malignancy	Entity	***n***
Benign	Lipoma	109 (76.8%)
	Spindle cell lipoma	1 (0.7%)
	Angiolipoma	4 (2.8%)
	Fibrolipoma	2 (1.4%)
Locally aggressive, not metastasizing	ALT	25 (17.6%)
Malignant	Myxoid LPS	1 (0.7%)

**Fig. 4:**
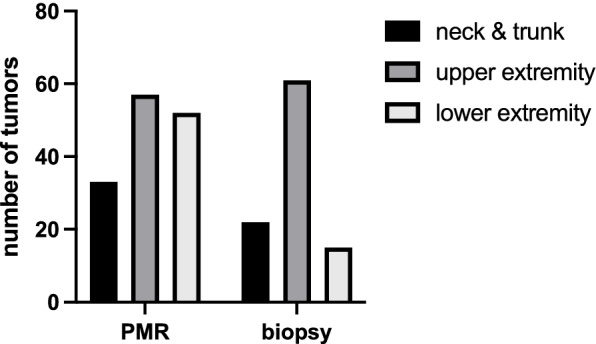
The anatomical distribution of the tumors was almost equal, but biopsies are favored in the upper extremity

Regarding the tumors that were treated by primarily marginal resection (PMR), 33 tumors were located in the neck and trunk, 57 were in the upper extremities, and 52 were in the lower extremities (Fig. 4).

Patients who received a biopsy of the lesion were on average 57.5 ± 17.9 years old. Thirty-six women and 62 men were in the biopsy cohort (Fig. 3). All biopsies were performed as open incisional biopsies and led to a histopathologic diagnosis. In 84 (86%) of the 98 lesions, the radiologist suspected malignant neoplasia. Sixty-seven (68%) malignant tumors were confirmed in the histopathological analysis. Seventeen (17.5%) lesions were benign, and 14 (14.5%) were ALTs. The exact entities of the lesions are listed in detail in Table 2. All but five tumors were later resected by required oncologic margins. Comparison of the histopathologic diagnosis from the biopsies (*n* = 98) to the histopathologic result after analysis of the complete tumor (*n* = 93) showed no change in diagnosis and a diagnostic accuracy of incisional tissue biopsy of 100%.

**Table 2:** Histologic diagnoses of the primarily biopsied lesions. No differences were observed in the histopathologic diagnosis from the incision biopsy in comparison with the analysis of the complete resected specimen

Malignancy	Entity	n
Benign	Lipoma	7 (7.1%)
	Spindle cell lipoma	2 (2.0%)
	Angiolipoma	2 (2.0%)
	Fibrolipoma	3 (3.1%)
	Hibernoma	2 (2.0%)
	Lipoma arborescens	1 (1.0%)
Locally aggressive, not metastasizing	ALT	14 (14.3%)
Malignant	Myxoid LPS	35 (35.8%)
	Pleomorphic LPS	24 (24.6%)
	Dedifferentiated LPS	6 (6.1%)
	Not-other specified LPS	2 (2.0%)

In 14 cases, a tumor was biopsied, which was rated as benign or intermediate by the radiologist. The decision to do a biopsy instead of a PMR was made after the MR images have been viewed by a musculoskeletal surgeon. The reasons were doubts about the benign character of the lesion by the surgeon or the patient’s explicit wish for a biopsy. In these 14 cases, the biopsy lead to the histopathologic diagnosis of the following entities: lipoma arborescence (1×), hibernoma (2×), lipoma (2×) spindle cell lipoma (1×), and ALT (3×), pleomorphic LPS (2×), and myxoid LPS (3×).

The lipomatous tumors were distributed throughout the whole body (Fig. 4). In the biopsy cohort, 22 tumors in the neck and trunk were biopsied before resection. In the same way, treated were 61 tumors in the upper extremity and 15 tumors in the lower extremity.

**Fig. 5:**
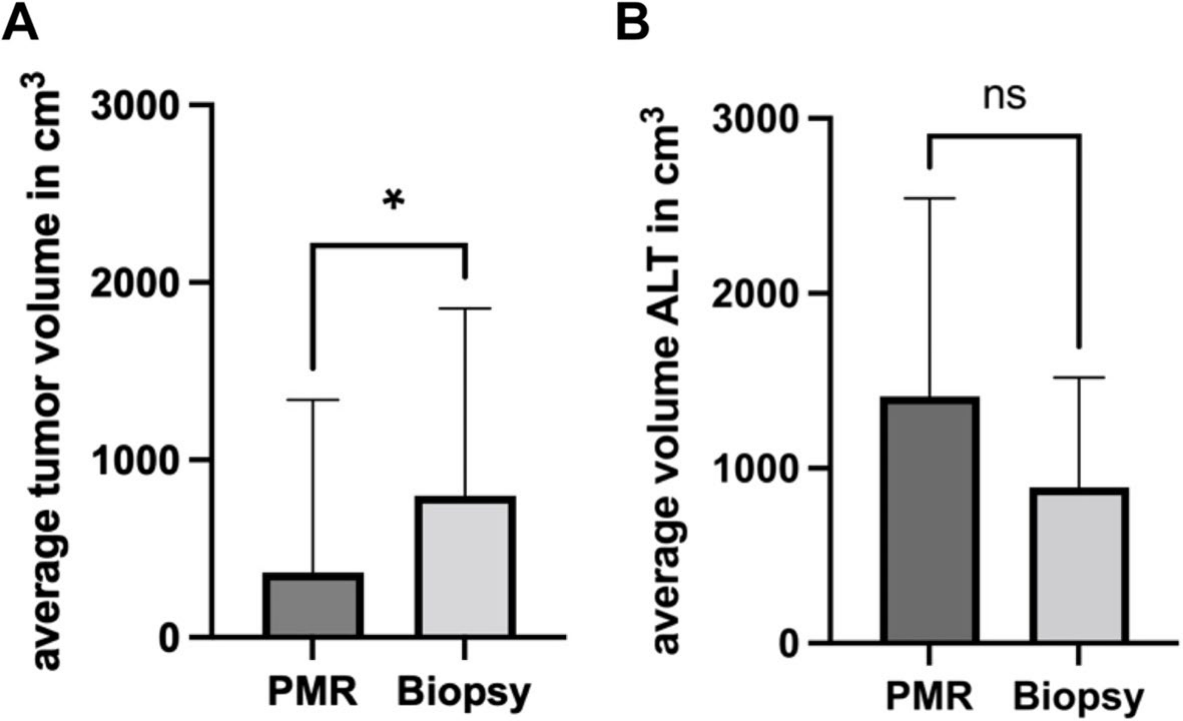
**A** Average tumor volume measured on MRI scans. **B** Average tumor volume of atypical lipomatous tumors (ALT). PMR, primarily marginal resection of the tumor; *indicates significant differences with *p* < 0.046; ns, not significant

The mean volume of the primary, marginally resected tumors in pathology was highly significantly smaller (566 ± 974 cm^3^) than the volume of tumors that were chosen for tissue biopsy first (799 ± 1055 cm^3^; *p* < 0.0001) (Fig. 5a).

When considering ALTs separately, ALTs that were chosen for PMR had an average volume of 890.9 ± 628.8 cm^3^ in MRI. In contrast, ALTs that were biopsied before resection had an average volume of 1410.9 ± 1133.8 cm^3^ in MRI (Fig. 5b). The difference was not statistically significant (*p* = 0.197). In both cohorts, ALTs were located in similar regions — for PMR, neck/trunk 2, upper extremity 5, and lower extremity 18 — for biopsy, neck/trunk 1, upper extremity 1, and lower extremity 12.

## Discussion

Due to the low incidence of soft tissue sarcomas, radiologists are rarely confronted by them [1]. This makes their differentiation from other lipomatous STTs even more difficult. Thus, musculoskeletal surgeons need to view MR images themselves and decide whether to marginally resect the tumor primarily or to biopsy it first. In cases of doubt or technical difficulties to perform a surgical biopsy, an a priori presentation of the case in an interdisciplinary tumor conference can be helpful.

The study’s data suggests that there is a trend towards biopsy for tumors with large volume. One must keep in mind that the tumor’s volume is unrelated to its dignity. Even larger lipomatous tumors with homogeneous appearance in MRI should be considered for PMR.

There are various guidelines with diagnostic and therapeutic algorithms for soft-tissue tumors, which are frequently followed insufficiently, especially in nonspecialized hospitals [18, 29, 30]. As MRI is the most important tool for diagnostic imaging, imaging guidelines are presented from a radiological point of view. In cases of radiological signs of malignancy and/or indeterminate lesions, tissue biopsy is recommended [31, 32].

From the surgeon’s point of view, a tumor with potential to local recurrence after incomplete resection requires identical treatment like a benign tumor in cases of lipomatous tumors because marginal resection of the tumor with its pseudocapsule is sufficient in both lipoma and ALT. Radical wide oncological resection is reserved for high-grade malignant tumors (G2 and G3 gradings). Therefore, radiological guidelines for soft tissue tumors and their adherence might not be adequate in cases of lipomatous tumors. Although diagnostic algorithms that are presented (especially recommended biopsies) are valid for most soft tissue tumors, they should be questioned in lipomatous tumors even if signs of malignancy might be present.

Coran et al. reported a correct detection rate of malignant LPS of 100% in 54 cases by radiologists in MRI [19]. In our reported findings, only one LPS was misinterpreted as an intermediate lesion. But 14 biopsies were performed of tumors, which had been rated as benign or intermediate by the radiologist. The reasons were doubts about the benign character of the lesion by the surgeon or the patient’s explicit wish for a biopsy. Out of these 14 tumors, five were malignant LPS.

We recommend primary marginal resection for small (< 3cm), epifascial lesions and deeper or larger lipomatous tumors with a truly homogeneous appearance in a T1-weighted sequence and with only very narrow or without septa [2]. The surgeon who performs the biopsy should be experienced in musculoskeletal tumor surgery and be prepared to do the final resection as well [33]. If a biopsy is needed, it should be an incisional biopsy, and it should be undertaken under general or plexus anesthesia. There is a tendency in the literature towards the use of core needle biopsies [26]. Core needle biopsies reveal the correct diagnosis in 68 to 84.9% of sarcoma patients, depending on the literature [34–36]. The rate of accuracy might even be higher in the combination of a fine needle aspiration biopsy [37], but incisional biopsy remains the gold standard [38, 39]. Our data supports this approach since all 98 biopsies produced a reliable histopathologic diagnosis. The surgical approach to the tumor is of utmost importance. It should be chosen with care, and it should always be kept in mind that further resection may eventually be necessary [39]. The surgical approach should be as direct as possible without crossing other compartments to avoid any tumorous contamination. If drainage is used, it should be let out through the wound.

All surgical specimens must be marked according to their anatomical location and sent to pathology for histological analysis [40]. After pathological analysis, all cases with malignant tumors should be discussed in an interdisciplinary tumor conference after radiological tumor staging of the patient [15]. Complete resection should always be favored in all tumors [41]. Each wrong diagnosis could bear extreme consequences for the individual patient.

All general limitations of a retrospective analysis apply to this study. Resolution of the MR tomographs (slice thickness and magnetic field strength) was not considered since they were not completely reported. Surgical treatment was performed by two surgeons, but a heterogeneous group of radiologists and pathologists performed imaging/tumor analysis.

## Conclusions

Our study shows that a very exact prediction of the tumor’s entity can be given with diagnostic support from MRI. Improved MRI quality allows an exact evaluation of tumor size, location, and depth within the tissue and a provisional diagnosis of the tumor’s entity. Therefore, we suggest a four-eye concept: when the radiologist assesses the tumors degree of malignancy as benign or intermediate. An experienced musculoskeletal surgeon views the MR images again. When the tumor appears lipomatous and truly homogeneous, it can be chosen for PMR. This results in faster and more comfortable treatment for the patient, especially with lipomas or ALTs.

However, the data supporting these results was retrospectively collected in one specialized institution. Prospective, multicenter studies are needed with an analysis for intra-observer and inter-observer comparisons, to recommend a true change in the standard of care for soft tissue tumors.

## Data Availability

The raw data are available upon reasonable request from the corresponding author.

## Abbreviations

ALT: Atypical lipomatous tumor
FISH: Fluorescence in situ hybridization
MDM2: Mouse double minute 2
MRI: Magnetic resonance imaging
LPS: Liposarcoma
PMR: Primary marginal resection
STT: Soft tissue tumor
WDLPS: Well-differentiated liposarcoma
